# Using EMG Biofeedback to Restore Closed-Loop Neural Control on a Powered Prosthetic Ankle

**DOI:** 10.1109/TNSRE.2026.3691653

**Published:** 2026

**Authors:** Brendan Driscoll, Joshua R. Tacca, Kasey Preisser, Ming Liu, Jonathan Stallrich, He Huang

**Affiliations:** Department of Electrical and Computer Engineering, North Carolina State University, Raleigh, NC 27606 USA.; Lampe Joint Department of Biomedical Engineering, North Carolina State University, Raleigh, NC 27695 USA; Lampe Joint Department of Biomedical Engineering, University of North Carolina at Chapel Hill, Chapel Hill, NC 27599 USA; Department of Statistics, North Carolina State University, Raleigh, NC 27695 USA.; Lampe Joint Department of Biomedical Engineering, North Carolina State University, Raleigh, NC 27695 USA; Lampe Joint Department of Biomedical Engineering, University of North Carolina at Chapel Hill, Chapel Hill, NC 27599 USA; Department of Statistics, North Carolina State University, Raleigh, NC 27695 USA.; Lampe Joint Department of Biomedical Engineering, North Carolina State University, Raleigh, NC 27695 USA; Lampe Joint Department of Biomedical Engineering, University of North Carolina at Chapel Hill, Chapel Hill, NC 27599 USA

**Keywords:** Electromyography, myoelectric control, haptic feedback, powered ankle prosthesis, lower-limb amputees

## Abstract

This paper aims to present that biofeedback based on electromyography (EMG) can help transtibial amputees to improve their capability to manipulate powered prosthetic ankles using Direct Electromyography (dEMG) control. First, we constructed a novel haptic based EMG biofeedback system composed of a HD haptic vest and an encoder that mapped EMG magnitude of residual shank muscles to tactile vibration patterns. Then we recruited six transtibial amputees and six non-disabled participants to conduct a position matching task, in which they conducted dEMG control on a powered prosthetic ankle with/without the biofeedback system after a brief acclimation. Impact of the EMG biofeedback system was evaluated based on their accuracy in matching specific targets in the position matching task. Our results demonstrated that 1) the EMG biofeedback can improve participants’ target matching accuracy; 2) the biofeedback led to more separable EMG signals among amputee participants when they tried to reach different targets; 3) for amputees, the improvement is more clearly observed when matching towards dorsiflexion targets; and 4) high performance variation among amputees is observed. Our work also demonstrated the feasibility of providing effective proprioceptive feedback based on muscle contraction level without involving any surgical procedure on lower limb amputees.

## I. Introduction

Individuals with lower limb amputations often rely on prosthetics to restore basic mobility. Traditional passive prosthetic legs are incapable of generating active power at the prosthetic joints, which leads to unnatural gaits and long-term challenges, such as chronic back pain [1]. Advanced robotic prosthetic legs have shown great potential to restore normative gait in users by providing controllable, active mechanical power around the joint [2]. However, the benefit of these robotics devices have not been consistently shown [3], [4]. One of the potential reasons is that these robotic devices, controlled by an independent computer, do not fully coordinate with the amputee users in locomotion [5], [6].

One solution to enable seamless coordination between human and robotic prosthetics is to remove the computerized prosthetic controllers and let the human nervous system drive the prosthetic device directly. A commonly used approach to achieve this is called direct electromyography (dEMG) control [7], in which the mechanics of prosthetic joints are driven based on the amplitude of residual antagonistic muscle activity. dEMG has been widely used for upper limb prostheses [7], but it is still a relatively new concept for lower limb prosthetics [8], [9], [10], [11], [12]. The related research has mainly focused on individuals with transtibial amputations to control robotic ankle prosthesis intuitively. Often, EMG activity recorded from the residual *tibialis anterior* (TA) was used to drive the ankle dorsiflexion (DF); and EMG recorded from the residual *gastrocnemius* (GAS) was used to drive plantarflexion (PF).

Direct EMG control has shown potential to improve gait [9], [11], [13], [14] and postural control [15] for amputees. Nevertheless, research has also shown that some transtibial amputees have difficulties in activating and coordinating residual antagonistic muscles [13], which is critical in dEMG control of robotic prosthetic legs [12], [16]. This challenge may be partly caused by the loss or diminished muscle proprioception after limb amputation, which impairs amputees’ ability to estimate their muscle contraction level. Evidence, such as that reported in [13], has shown that amputees need visual feedback of muscle activation level in order to generate the expected EMG control signal to control a robotic ankle prosthesis during locomotion. Hence, a method that can restore the prosthesis user’s muscle proprioception on the residual limb is needed to improve the capability of amputees in dEMG control of robotic prosthesis legs.

Sensory feedback for individuals with limb loss to enhance neural control of prosthetics has been investigated intensively; however, the majority of research has focused on upper limb amputees [17], [18]. While there have been studies focused on sensory feedback for lower limb amputees, most of these studies used passive prosthetic devices and did not pair sensory feedback with any dEMG control for closed-loop operation of robotic prostheses [19]. Researchers have used electrical stimulation of peripheral nerves or the spinal cord to evoke natural sensations of the missing limb in individuals with lower limb amputation [20], [21]. These nerve stimulations elicited somatotopically matched haptic sensation in the missing foot and leg. A noninvasive version is also reported in [22]. In these studies, plantar pressure data were recorded using insole pressure sensors and then encoded into stimulation patterns delivered to the nerves. When this sensory feedback was provided, amputees demonstrated improved locomotion balance and reduced cognitive load [20], [23], [24]. However, it is noteworthy that direct nerve stimulation cannot reliably evoke proprioception [25]. Moreover, all participants in these studies used their own daily passive prosthetic devices, and no neural control of the prosthesis was implemented.

Recently, a group demonstrated closed-loop neural control of a robotic ankle prosthesis based on a surgical technique called agonist-antagonist myoneural interface (AMI) [26], [27]. The AMI surgically connects the tendons of residual agonist-antagonist muscle pairs [28] so that contraction of one muscle stretches its counterpart, restoring proprioceptive feedback of the antagonistic muscle state [26]. Combined with dEMG control, AMI enables bidirectional neural signaling and closed-loop prosthetic operation, which improve preferred walking speed, terrain adaptation [14], and joint control accuracy [29]. However, the major drawback of AMI lies in its invasive nature. Participants need to go through a surgical procedure that carries potential risks such as infection [30]. Moreover, many lower limb amputees, whose amputations are often resulted from diabetes-related complications, may not be eligible due to their impaired capability for wound healing [31], [32].

Hence, we are interested in exploring an alternative, non-invasive approach to provide muscle proprioceptive feedback and evaluate how feedback can enhance the user’s ability to drive a lower limb robotic prosthesis via dEMG control. Sensory substitution is a potential non-invasive solution, which has been widely studied for closed-loop control of upper limb prostheses [33]. Often, the contact pressure or force applied to the prosthetic hand was fed back to the user via an intact sensory modality, such as visual, auditory, or haptic feedback. Haptic feedback is the most commonly used approach as it does not distract from visual or auditory attention needed for other daily tasks [34]. Haptic feedback can be delivered through either vibrotactile or electrotactile actuators attached on the skin surface [35]. Another approach is to use sensory substation to feed back the activity of residual muscles (i.e., EMG biofeedback). Since the EMG signals from the residual muscles are also the prosthesis control signals, EMG biofeedback allows prosthesis users to produce better grasping and force steering task performance than when hand contact force was used as feedback [36], [37]. Further studies showed that since this feedback is based on intermediate signals generated by the amputee, it can lead to a stronger internal model with reduced uncertainty in the forward pathway for predictive control [36]. Additionally, relying on EMG biofeedback, rather than the feedback of device motion or contact force, reduces the feedback delay and allows for improved motor control capability [17].

Inspired by the previous research, the objective of this study is to use EMG biofeedback via tactile sensory substitution to enable closed-loop EMG control of a robotic ankle prosthesis. As the first step to evaluate the effects of EMG biofeedback, we recruited non-disabled (ND) participants and participants with transtibial amputations (TB) to drive a robotic prosthetic ankle using dEMG control in a position matching task with and without EMG biofeedback. The results may inform a novel, non-invasive wearable feedback system to enable closed-loop operation of robotic prosthetic legs to achieve seamless coordination between human and robotic device for improved daily function for individuals with lower limb amputations in the future.

## II. Methods

### A. Participants.

With IRB approval (NC State University IRB approval protocol number 24436) and signed informed consent, we recruited 6 participants with transtibial amputations (TB01–06). TB participants’ demographic information is summarized in Table I. Additionally, 6 participants with no disabilities (ND) were recruited as a means of providing a baseline on how accurately unimpaired individuals can complete the position matching task. ND participants consisted of 2 females and 4 males, ages ranging from 21–30, an average weight of 72 ± 15 Kg, and an average height of 1.7 ± 0.11 m. All participants possessed no history of cognitive, visual or auditory impairments that could have impacted on their ability to participate, neither did they possess any history of other neurological injuries or disorders.

### B. EMG Biofeedback Setup.

In this study we adopted a haptic feedback system developed in our previous work [38]. While key information about the system will be provided here for completeness, more detailed discussions can be found in [38] and [39]. This approach provides feedback through a grid of vibrotactile motors, which previous work has demonstrated to be effective for delivering precise and intuitive haptic feedback [40], [41].

A high-density haptic vest (bHaptics Tactsuit x40, bHaptics Inc., Daejeon, South Korea) was used as the feedback interface for this study. Haptic sensation was provided through the two 4×5 arrays of individually programmable vibrotactile motors. This interface was used to communicate the amplitudes of the control signals, which are used to drive the prosthetic ankle for dorsiflexion and plantarflexion respectively.

We applied the perceived position encoding (PPE) strategy to generate needed biofeedback signal. This strategy is designed to take advantage of the high-density haptic grid of the vest [38]. The approach works by activating multiple adjacent motors to generate a phantom sensation of a single vibrator between the two motors [42]. The perceived position of this phantom vibrator was used to represent the amplitude of the EMG activity from the TA and GAS muscles. The EMG amplitude of the TA was displayed on the front of the vest and the GAS on the back, with the phantom vibrator moving from the bottom of the vest to the top as the EMG amplitude increased. The locations of the phantom vibrators hold a linear relationship with the respective EMG amplitudes.

**Fig. 1.**
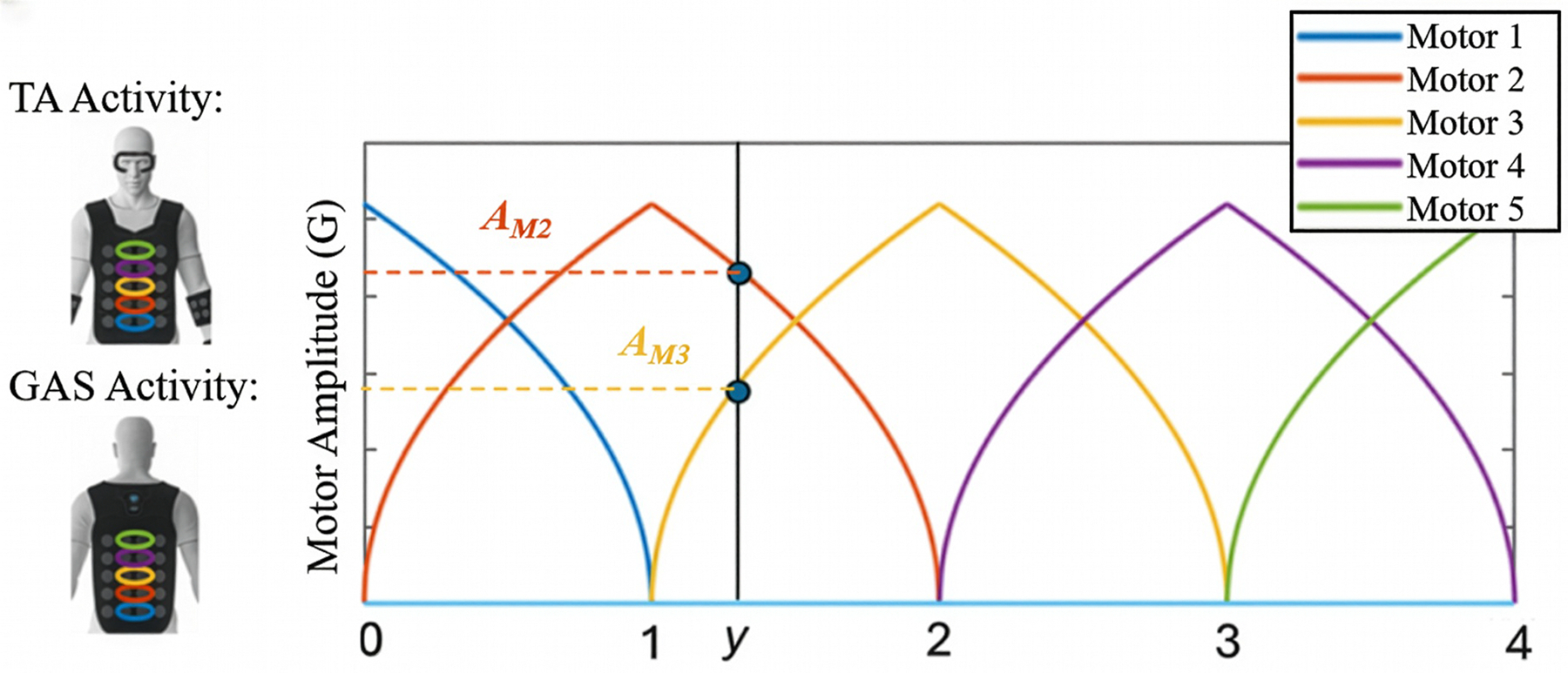
Diagram of haptic feedback encoding strategy, where y represents an example of scaled muscle activities (EMG amplitude). *A*_*M*2_ and *A*_*M*3_ are the amplitude of the motor row 2 and row 3 respectively. This encoding strategy was applied simultaneously on the front of the vest to give biofeedback on TA activity and the back of the vest to reflect GAS activity.

The phantom vibrator appeared in the middle two columns of each vibrator array. The two adjacent motors in each row behaved identically to one another to create the sensation of the phantom vibrator moving along the center of the array. Using the bottom row of the array as the origin, the location of the phantom actuator was calculated using Eq. 1.

(1) $$y=\frac{\left(v\;-\;v_{off}\right)}{MVC\;-\;v_{off}}\times 4$$

where v was the amplitude of the EMG control signal used to control the prosthetic ankle, $v_{off}$ was the initial offset, which represents muscle activity when the participants are fully relaxed. MVC (Maximum Voluntary Contraction) was the maximum muscle activity the participants can achieve. The five rows of motors are located in the same coordinate system and are labeled based on its location $M_{n}$, n=0,1,...,4. To generate the perceived vibration amplitude at y, the vibration amplitudes of the motor rows labeled by n=floor(y) and n=ceil(y), $A_{M_{n}}$, can be set as:

(2) $$A_{M_{n}}=P\sqrt{1-|y-n|}$$

where P was a constant. An example of this encoding approach was shown in Fig. 1.

### C. Equipment Setup and Data Collection.

#### 1) Powered Prosthetic Ankle:

The powered prosthetic ankle for this study used pneumatic artificial muscles (PAMs) to produce torque and move the ankle joint [43]. Two PAMs were positioned in front of the ankle to serve as the artificial TA muscle, and two more in the back of the device to serve as the GAS muscles. The activities of the corresponding muscles controlled the torque produced at the ankle joint by manipulating the pressure in the PAMs through proportional pressure regulators (MAC 36 series valve and PPC36A Pressure Regulator, Wixom, MI, USA) with a range of 0 to 90 psi [44]. The pressure of each muscle scaled linearly with the EMG control signal, which ranged from 0–10V. The kinematics of the powered prosthetic ankle were recorded using a marker-based motion capture system (VICON, UK). Kinematic data was recorded at 100 Hz and passed through a 7Hz low pass filter for smoothing.

#### 2) Real-Time EMG Control:

To control the device, EMG signals were recorded and filtered into usable control signals sent to the pressure regulators, which controlled the internal pressure of the PAMs. EMG activity of TA and GAS was recorded in real-time at 1000 Hz (Motion Lab Systems MA-400, Lake Elsinore, CA, USA). For amputee participants, low profile electrodes (Neuroline 715, Ambu, Ballerup, Denmark) were adopted, so they can be integrated into TB’s sockets. For ND, bipolar electrodes (Norotrode 20, Myotronics, Kent, WA, USA) were used.

To calculate the EMG control signals, the EMG envelop of the GAS and TA were calculated in real-time using Simulink (Mathworks Inc., Natick, MA, USA) to define the amplitude of the muscle activities of TA and GAS separately. The raw EMG signals were high-pass filtered (100 Hz, 2^nd^ order Butterworth), then rectified, then passed through a low-pass filter (2 Hz, 2^nd^ order Butterworth). This filter was applied in order to remove motion artifacts and match the frequency bandwidth of the PAMs [43].

### D. Experimental Setup.

1. *EMG Placement:* muscles were located via palpation to find the TA and GAS muscles. For ND participants, the TA was identified at the lateral side of the Tibia, 8 cm below the tibial tuberosity. The GAS electrode placement was located by identifying the lateral muscle belly. For TB participants this same procedure was used, however since amputation can alter muscle anatomy [45] if the signal was not clear then electrodes for that muscle would be shifted, approximately 1 cm at a time, towards the muscle belly until the signal became isolated.
2. *EMG calibration:* MVCs were performed to measure the maximum EMG signal output from both the TA and GAS sensors. After the EMG electrodes are attached on the selected dEMG sites, the participants are instructed to “point your toes down” and “point your toes up” as hard as they can for 2–3 seconds and then relax. This was in order to get the maximum values for the GAS and TA amplitudes respectively. This procedure repeats 3 times for both muscles and the measured maximum muscle activities at the corresponding sites are used for the *MVC*s.
3. *TB Recalibration*: Recalibration for TB participants was conducted to address the practical challenges for amputees, on whom residual muscle co-contraction is very common. This procedure starts with identifying natural lines of co-activation in the control space defined by TA and GAS EMG amplitude as horizontal and vertical coordinates, respectively. To identify the natural lines of co-activation, the participants use a cursor controlled by dEMG to cover as large as an area in this control space following the procedure reported in [46], these natural co-activation lines are defined as the two vectors that bound the participant’s control space [16]. These vectors reflect the maximum capability of participants to isolate their DF and PF muscles [16]. We project the real-time filtered TA and GAS EMG signals along these natural lines of co-activation and use these projections as the control signals for the PAMs for dorsiflexion and plantarflexion, respectively. This approach ensured that all participants could generate separable PF and DF control signals even with high co-activation. An example of these co-activation lines can be seen in Fig. 7. When providing biofeedback, the feedback signal was directly proportional to this recalibrated control signal.
4. *Prosthesis calibration:* To ensure the prosthesis would return to neutral position when the participant was relaxed, an offset between 1.5–2.5 V was applied to the pressure regulators, which were connected to both pairs of PAMs. Then, the control signal was rescaled to ensure that the MVCs for the TA and GAS could generate a control signal equal to the maximum input (10V) of the pressure regulator. Once gains were set, the maximum range of motion (ROM) for DF and PF were defined as the angular distance between the neutral position and the maximum achievable angle for DF and PF, respectively. Going forward, we discussed ankle position as a percentage of the total ROM for DF and PF.

### E. Experimental Protocols.

A position matching task was used as the core procedure to quantify the accuracy of participants’ control over the robotic prosthetic ankle. As a well adopted test in rehabilitation and prosthesis control [26], position matching evaluates the capability of human participants to accurately orientate their body segments or assistive devices. Here we adopted this task to evaluate participants’ capability to orientate a powered prosthetic ankle through dEMG control.

To perform the position matching task, participants sat comfortably in a chair with the knee of the tested leg (amputated legs for amputees and right legs for ND) held at approximately 30 degrees. While seated, an illustrative command was displayed on a screen which prompted the participant to match the powered ankle’s orientation with the specified position by activating their muscles. Participants were instructed to move the ankle to the target position and hold it steady for one to two seconds, they would have 5 seconds to activate their muscles to attempt to match the target. After each of these matchings, the participant was instructed to fully relax to allow the ankle to return to the neutral position before the next matching occurred.

#### 1) Acclimation Trials:

**Fig. 2.**
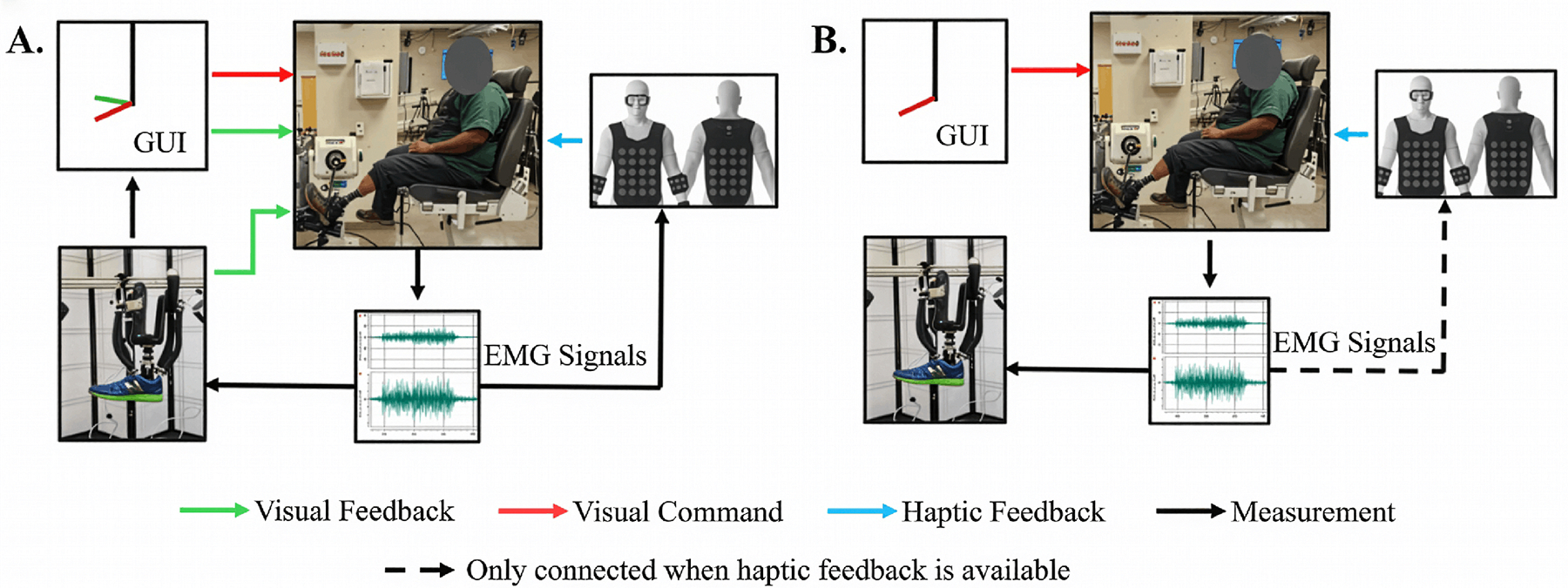
Experimental Protocols where A) shows the setup for acclimation trials, in which participants received 1) visual feedback on the current ankle position and 2) performance based feedback after reaching for each target besides haptic feedback. B) Shows the evaluation trial setup in which participants received no visual feedback and could only rely on haptic feedback.

To allow participants time to understand the prosthesis control, the haptic feedback, and how their muscle activations altered the position of the ankle, a series of acclimation trials were performed. As illustrated in Fig. 2A, participants performed a position matching task while receiving both haptic and visual feedback. The first source of visual feedback was the ankle itself, which participants could view during acclimation. The second was performance-based feedback which informed participants when they made a successful match and, if the match was unsuccessful, showed the actual ankle angle in relation to the target position to visualize error. Here, a position was considered “matched” if the participant held the ankle within ± 5% of the ankle’s DF or PF ROM of the target for 0.5 consecutive seconds (based on whether the target is a DF target or a PF target), otherwise the position was considered “unmatched”.

Acclimation trials were divided into sets of DF and PF trials, where the participant was prompted to move to either 25%, 50%, 75%, or 100% DF or PF, respectively. Each target was repeated 5 times for a total of 20 targets per trial. Participants performed DF and PF trials in a random order. Acclimation trials were repeated until the participants met or failed the completion criteria. To meet the completion criteria participants had to get 70% of targets in a trial “matched” in both a DF and PF trial, and they must have completed at least 3 DF trials and 3 PF trials. The participant would be considered to have failed the completion criteria if they could not get 70% accuracy in a trial after 10 attempts at the DF or PF trials. If they failed the acclimation trials, they would not be eligible to continue the study and be excluded.

#### 2) Evaluation Trials:

After acclimation, two evaluation trials were performed. One evaluation trial was done in the with biofeedback (BF) condition and another in the no biofeedback (NBF) condition, these were done in a randomized order. As illustrated in Fig. 2B, a display prompted the participant to move the ankle to one of 8 targeted positions, either 25%, 50%, 75%, or 100% ROM for DF or PF, respectively. During an evaluation, each target was repeated five times and total 40 targets are matched in a randomized order. For evaluation trials the robotic ankle was kept out of view, so that participants received no visual feedback of the ankle’s position, and participants were not informed whether they successfully matched the target or not after each matching.

### F. Data Analysis.

Although the participants are instructed to hold the position when they reach the given target, it was impractical to assume that they can maintain a fixed position for long period of time. In most situations, the participants move the ankle to a position and then drift around it. To quantify the control performance, we defined the concept of Patient’s Selected Position (PSP). During evaluation trials, we consider that participants have selected a position if 1) the ankle was held in place away from the neutral position by at least 5% ROM (DF or PF accordingly); 2) its angular velocity below 10% ROM per second for at least 0.5 second. If there was more than one time span, in which the two conditions are met in a matching effort, the longest time span was used. The average ankle angle in this time span was used as the PSP.

#### 1) Median Absolute Error (MAE):

To quantify the accuracy of each participant when moving to specific targets, the absolute error was calculated as the absolute difference between the target and the PSP. Since each evaluation trial required participants to move to each target 5 times, the MAE was averaged from these 5 observations for each target in each condition. MAE was used instead of other error metrics such as RMSE as MAE is known to be robust towards outliers which may bias the results [47].

#### 2) Quantifying Separation of Muscle Activity for Different Targets:

To understand how consistent muscle activations were generated to hit a given target, we used the silhouette scores of the EMG signals to quantify the EMG separability. A common tool to evaluate clustering quality, silhouette scores quantify the cohesion and separation among observations corresponding with different clusters. A positive silhouette score close to 1 indicates high similarity among each observation in a given cluster and a distinct separation from neighboring clusters. This would show that participants could generate accurate and distinct EMG signals for each given target. A negative silhouette score close to −1 would indicate an inability to generate distinct clusters for different targets, showing that participants were unable to accurately control the ankle or distinguish between targets. This metric allows us to further explore how biofeedback may have influenced the performance of individual participants.

To analyze the EMG data, the raw EMG signals were demeaned, bandpass filtered (50–350 Hz), rectified, and then passed through a 4th order low-pass Butterworth filter (6 Hz). This filter differs from the filter used for the dEMG control signal in that it retains more of the original EMG signal while maintaining a smooth signal for qualitative analysis [15], [46]. Next, we calculated the silhouette scores using two EMG features, the average amplitudes of the filtered TA and GAS signals during the time span when the PSP was calculated. We classified clusters based on the targeted positions for a total of 8 different clusters. Detailed procedures on this calculation are presented in the supplementary document.

### G. Statistical Analysis.

We expected that participants would perform more accurately in the position matching task while receiving biofeedback compared to when no biofeedback was provided, as evidenced by a lower MAE across the different targets. The MAE acted as the dependent variable in our statistical analysis, and the key independent variables included: feedback condition (BF and NBF), and target position.

Additionally, we expected that TB participants would improve their dEMG control by producing better separated EMG signals for the different targets when receiving feedback. To test this hypothesis, we used median silhouette scores as our dependent variable, with feedback condition as the independent variables.

To test our prediction, we used two linear mixed effects models (LME) to evaluate both fixed and random effects. One LME was run on the ND participant data and the other one on TB participant data. Both LMEs had categorical fixed effects of target positions and feedback conditions with random intercepts included for each participant to account for individual differences in performance. To find any effects from target position, the MAE at each target was compared to the MAE at the 100% PF target as a baseline. To confirm any significant differences in MAE between different targets, pairwise comparisons were used with the Sidak correction for multiple comparisons [48]. Models were fit using R (v4.3.0) using the “lme4” package. Interactions and effects were deemed significant at P < 0.05. Here LMEs are adopted to replace the more common repeated ANOVA due to the expectation of high intersubject variation [49]. A further post-hoc comparison was conducted to test whether BF significantly reduced MAE when compared to the NBF condition (one-tailed paired t-test) at P < 0.05.

To prevent outliers from driving the data, an inclusion/exclusion criterion was used to determine if any participant’s data should be excluded from the LMEs. For this study, the EMG silhouette score was used to determine eligibility. This was because a negative median silhouette score would indicate a general inability to generate distinct EMG signals to reach the different targets required for the experiment. A participant would be excluded from the LME if they were unable to get above a median silhouette score of 0 for either of the evaluation trials.

## III. Results

### A. Position Accuracy.

#### 1) Average Position Matching Performance:

**Fig. 3.**
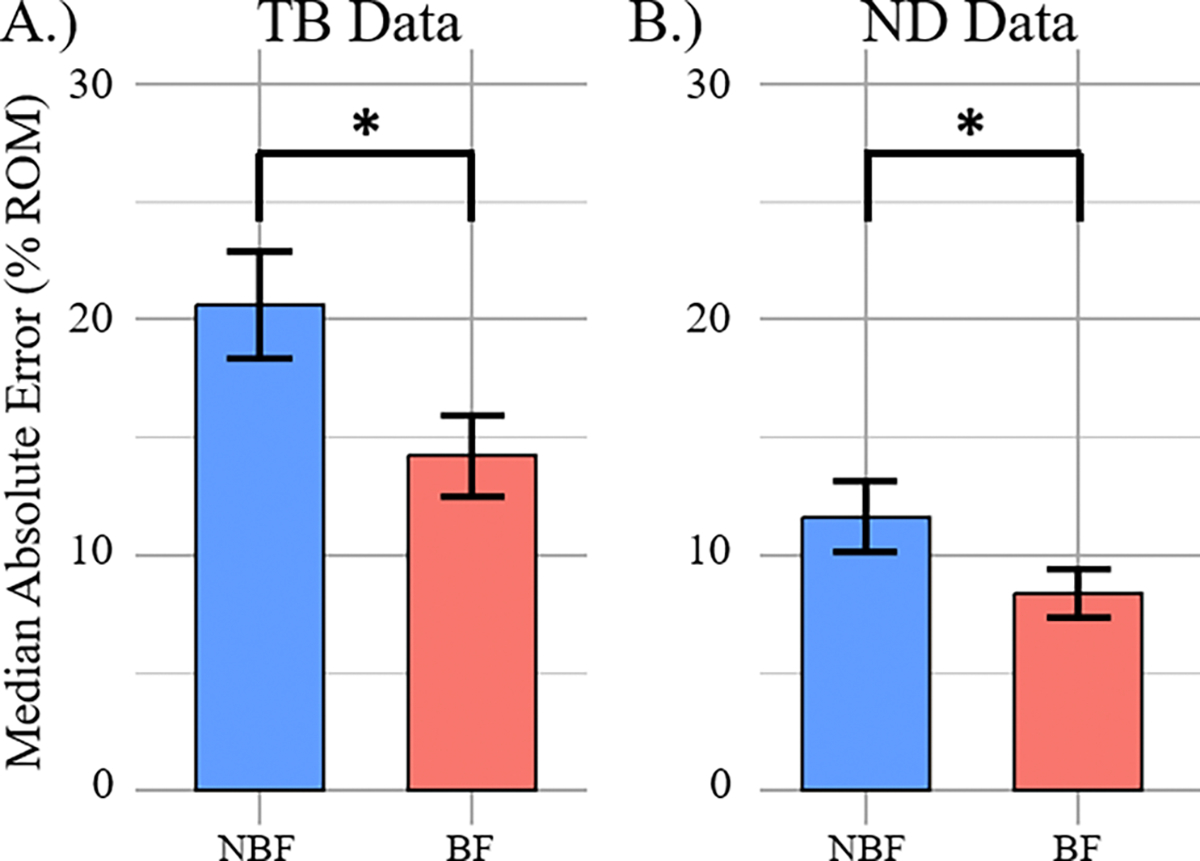
Averaged median absolute error (MAE) in the ankle position matching task. The results are derived from (A) TB participants and (B) ND participants. Blue bars and red bars represent the without biofeedback (NBF) and with biofeedback (BF) conditions respectively. The symbol ‘*’ denotes a significant effect of feedback condition found by the LME models at P < 0.05. TB06 was excluded as an outlier.

Fig. 3. Shows the mean and standard error of the MAE for the TB and ND groups in the NBF and BF conditions. Both groups were able to perform the position matching task with less errors in the BF condition where EMG based biofeedback was provided. This improvement in target matching accuracy was found to be significant by the LMEs, which detected a significant effect of feedback condition. The TB group had an estimated 6.44% ROM (DF or PF corresponding to the targets) lower MAE during the BF condition than the NBF condition (SE = 2.46%, P = 0.011). For the ND group, the BF condition had an estimated 3.28% ROM (DF or PF corresponding to the targets) lower MAE than the NBF condition (SE = 1.64%, P = 0.048). This shows that both groups clearly benefited from using the biofeedback in the position matching task.

#### 2) Performance Across Targets:

**Fig. 4.**
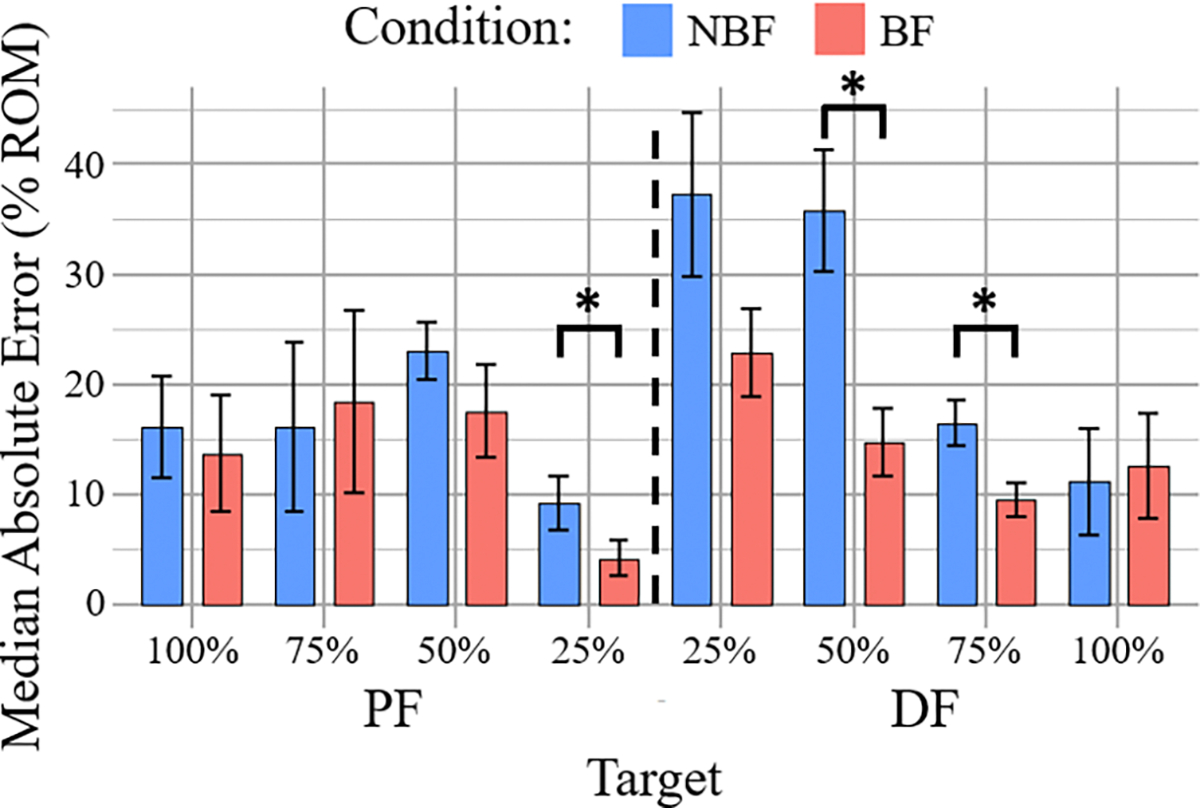
TB performance in posture matching task presented as the average median absolute error (MAE) across all different targets. Blue bar and red bar represent the without biofeedback (NBF) and with biofeedback (BF) conditions respectively. The symbol ‘*’ denotes targets for which significant improvement was identified after the biofeedback was provided. TB06 was excluded as an outlier.

Fig. 4. further explores the performance of the TB group by showing the error under two feedback conditions across each of the DF and PF targets. This figure shows that the biofeedback was most helpful for DF targets, particularly the 75% and 50% DF targets. Significant performance improvement was also observed in 25% PF target.

The LME found that 25% and 50% DF had significantly higher errors when compared to the baseline (100% PF). The 25% and 50% DF targets had an estimated 15.16% and 10.34% DF ROM greater MAE than the baseline of 100% PF (SE = 4.92%, P = 0.003; and SE = 4.92%, P = 0.039), respectively. Performing pairwise comparisons with the Sidak correction confirmed that 25% DF had significantly higher MAE than multiple other targets, including 23.35% greater MAE than 25% PF (P < 0.001), 17.1% greater MAE at 75% DF (P = 0.025) and 18.21% ROM greater MAE at 100% DF (P = 0.016). This also showed that errors at 50% DF had an estimated MAE 18.5% ROM greater than 25% PF (P = 0.022).

These results show how TB participants had the greatest difficulty when matching these DF targets. Paired t-tests between the BF and NBF conditions at each target reveal that performance was significantly improved in the BF condition for the 50% and 75% DF targets, at P = 0.029 and P = 0.038 respectively, as well as one PF target at 25% PF (P = 0.037). This suggests the biofeedback was most helpful at these DF targets, as well as the 25% PF target.

The ND group performed much more consistently across the different targets. While the LMEs did find a significant effect of target in the ND group with the 75% DF target having an estimated 7.04% ROM greater MAE than the baseline of 100% PF (SE = 3.28%, P = 0.035), performing the Sidak correction for multiple comparisons revealed no significant differences in MAE between targets.

#### 3) Silhouette Scores:

**Fig. 5.**
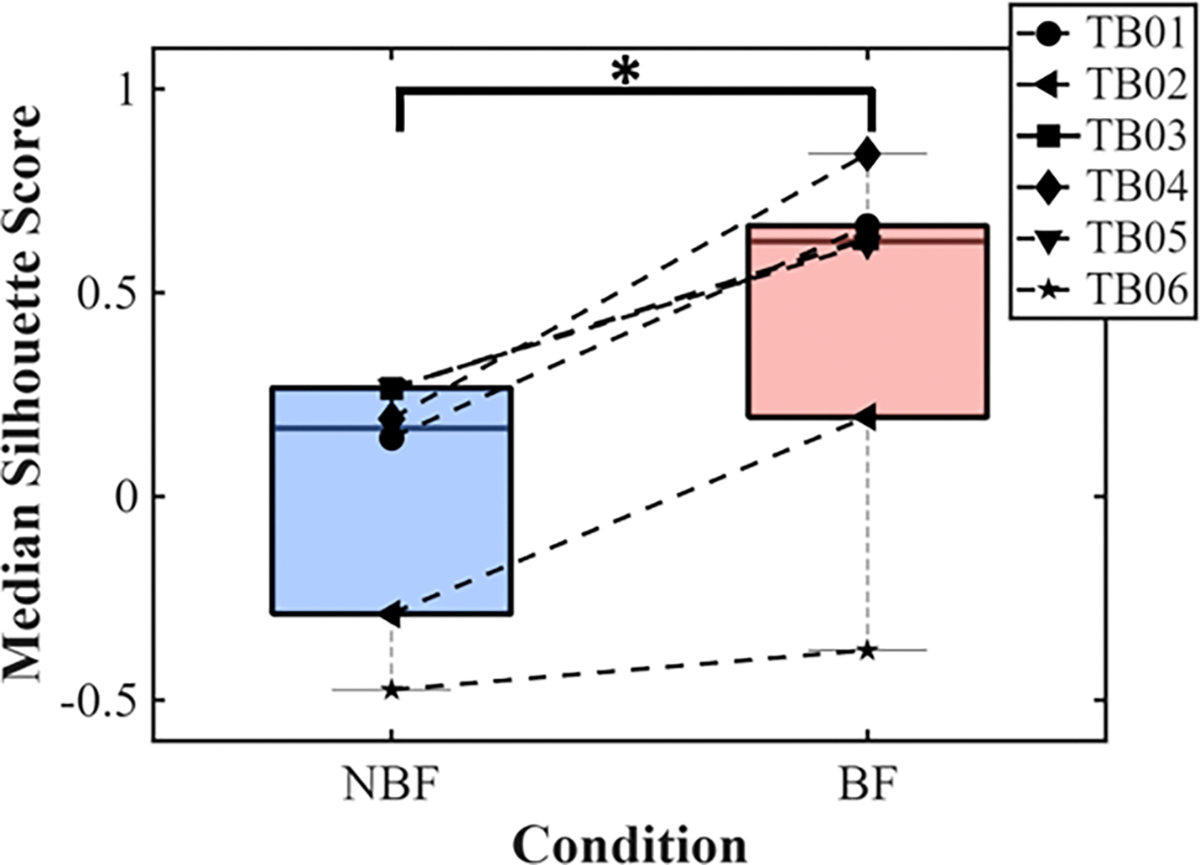
Boxplots showing median silhouette scores of TB participants for NBF and BF conditions. The center bar represents the median silhouette score among participants, the top and bottom edges show the 25^th^ and 75^th^ percentiles respectively, and the whiskers denote the minimum and maximum values. The symbol ‘*’ denotes a significant effect of feedback condition found by the LME model at P < 0.05. TB06 data was included.

Fig. 5. Shows the TB group’s silhouette scores, calculated from the TA and GAS amplitudes. This was used to quantify TB participants’ ability to generate distinct control signals for different targets. From Fig. 5, we can see that TB participants consistently achieved higher silhouette scores in the BF condition compared to the one in the NBF condition. This improvement in silhouette scores was confirmed by the LMEs which found a significant effect from feedback condition, with silhouette scores estimated to increase by 0.288 in the BF condition when compared to the NBF condition (SE = 0.125, P = 0.024). These results show that under the BF condition participants achieved better EMG separability. Fig. 5. also shows that there was one TB participant that failed to meet the inclusion/exclusion criteria, with TB06 having a negative median silhouette scores in both conditions. This indicates that TB06 was unable to effectively complete the position matching task under either condition, so the participant was treated as an outlier, and their data was excluded from the LME models.

#### 4) Individual Performance:

**Fig. 6.**
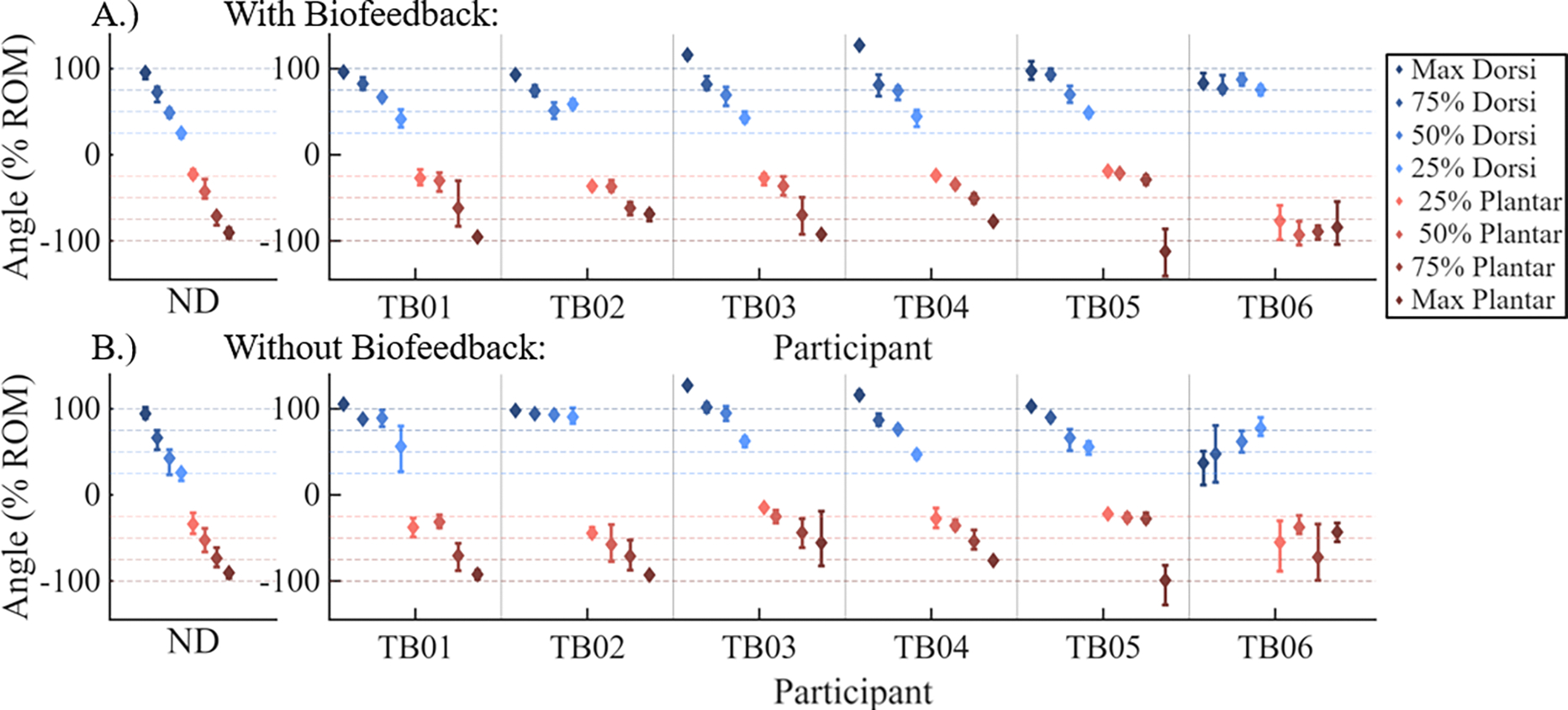
Participant Selected Position (PSP) data for ND participants (average shown on far left) and TB participants for (A) the with biofeedback (BF) condition and (B) the without biofeedback (NBF) condition. The diamonds show the median PSP for each target and vertical lines show the 25% and 75% interquartile ranges.

Fig. 6. Provides further details on the performance of individual TB participants by showing the PSPs across each target. The average performance of ND participants is provided as a reference, which shows how ND participants were able to consistently generate PSPs near the target positions with minimal overlap with adjacent targets. This contrasts with the TB participants who showed high intersubject variability.

These results align with our findings in Fig. 4, where most participants appear to move their PSPs closer to the correct targets in the BF condition when compared to the NBF condition, particularly at 75% and 50% DF, and 25% PF. For the 75% DF target, 5 out of 6 participants improved accuracy with only TB05 not improving in the BF condition. Both the 50% DF and 25% PF targets saw 4 out of 6 participants improving accuracy in the BF condition, with the exceptions in both cases being TB05 and TB06.

#### 5) Outlier Performance:

**Fig. 7.**
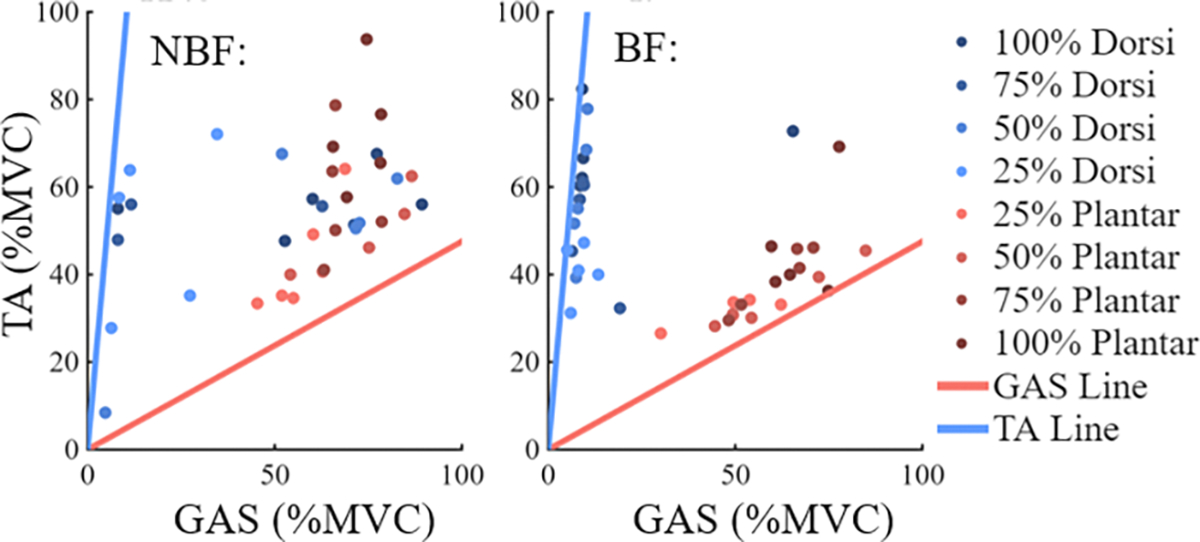
EMG data from TB06, where each dot represents the EMG control point achieved when the participant reached for the respective target. The red and blue lines show the co-activation lines found during recalibration for the TA and GAS, respectively.

Fig. 7 paints a clearer picture of TB06’s performance by showing the EMG data recorded from the evaluation trials. Under the NBF conditions, EMG control points, of which the coordinates represent the amplitude of the TA and GAS signals, appear randomly distributed in the area between the two natural co-activation lines found in the recalibration procedure. This indicates a large amount of co-activation, which shows that during this condition TB06 struggled with differentiating between DF and PF. Under the BF conditions, the majority of EMG control points are clustered close to the natural co-activation lines. Minimizing co-activation in this way is important for isolating the DF and PF motion, as co-activation causes the ankle to stiffen and move towards the neutral position.

## IV. Discussion

In this study, we presented a novel EMG biofeedback system, composed of a HD haptic vest and an encoder that mapped EMG magnitude of residual ankle muscles to tactile vibration patterns. Our system was combined with a dEMG controller that augmented the prosthesis users’ sensation regarding the activation levels of their antagonistic ankle muscles for closed-loop operation of a robotic ankle prosthesis. As the first step to evaluate this design, we asked our participants to control the prosthetic ankle in a position matching task with and without our EMG biofeedback system. The study showed that EMG biofeedback improved the participants’ control of their antagonistic muscles for more accurate dEMG control during the position matching task, compared to their control accuracy without EMG biofeedback. The improvement in DF control with biofeedback that was observed in this study may enable TB participants to have a better chance of avoiding foot drop, reducing the risk of trips and falls, and improving their ability and efficiency to navigate difficult terrain such as stairs or uneven ground [50]. In non-disabled populations, it has been observed that typical variability of foot motion creates the possibility of occasional scuffing with as little as a 2 degree change in swing ankle angle [51]. We found that with the biofeedback compared to without biofeedback, participants had 6.44% of range of motion lower error in the position matching task. This difference corresponds to about 2 degrees less error on average. Thus, we speculate that the improvement in position accuracy with biofeedback is functionally meaningful for TB amputees. Our results are consistent with previous research [29] which restored the sense of residual muscle proprioception through an invasive surgical procedure, i.e., AMI.

Our results further confirmed that providing proprioceptive feedback based on muscle contraction level can benefit users by improving the accuracy of their dEMG control signals for controlling an external machine. In addition, our designed wearable EMG biofeedback system provides a potential non-invasive solution for individuals with limb loss, who might not be eligible for AMI surgery or cannot afford the procedure, to enable or enhance their capability to operate dEMG-controlled robotic prostheses in the future.

While inspired by previous studies, the augmented feedback system developed in this work was unique compared to existing approaches. Specifically, we chose to restore proprioceptive feedback of muscle activity rather than foot plantar haptic sensation [19], [52] or ankle joint position [53]. Feedback of muscle activation offers advantages because it can support EMG-based prosthesis control across different dynamic conditions, whereas plantar pressure feedback or joint-position feedback is only relevant to limited aspects of ankle dynamics. For instance, in locomotion, ankle muscles primarily drive ankle position in the swing phase, whereas in the stance phase they modulate joint impedance and push-off torque rather than joint angle. Therefore, restoring the sensation of ankle position may only enhance performance during the swing phase. Similarly, haptic sensation under the prosthetic foot is limited to the stance phase and reflects not only the prosthesis state but also the terrain and load-bearing conditions. Thus, plantar pressure feedback may influence little on neural prosthesis control signals. To enhance the user’s ability to generate appropriate EMG signals for dEMG prosthesis control, we augmented the user’s perception to give a sense of how their own muscle activation affects the dynamics of the prosthesis. This approach established a closed-loop pathway that enabled appropriate coordination of antagonistic muscles to regulate prosthesis joint mechanics, as long as the user understood the mapping between muscle activity and prosthesis joint behavior after a brief acclimation. Although this study focused on a joint position matching task, the same paradigm can be extended to more functional tasks such as balance and locomotion.

Our biofeedback system enhances the user’s perception of their own muscle contractions rather than relying on signals specific to a particular device or control scheme. As a result, the feedback is inherently generalizable and can be applied to any dEMG-controlled prosthesis, regardless of the specific device or controller design. Future work could validate this assumption through testing the biofeedback system on different dEMG control strategies that have been used in literature, such as the impedance controller implemented by Stafford et al. [11].

We chose the HD haptic vest as the feedback interface for this study, because its large surface area allows for clear feedback delivered to the torso, which previous work has shown is intuitive for participants to understand [38]. Having a feedback interface that is separate from the prosthetic device itself may make this system inconvenient for wider clinical applications. Moreover, the vest is larger than other interfaces, such as haptic thigh bands [54] or waist bands [55]. The current setup fits more for a clinical training tool instead of a system which is activated around the clock. In the future, a feedback interface built into the prosthetic socket, like that proposed in Barontini’s work [56], will be more appropriate for take home use.

Another potential challenge is sensory adaptation, where the stimulus is eventually ignored by the user [57] after being exposed to continuous haptic stimulus for a long period of time. This is a practical challenge for all haptic biofeedback systems. For the prosthesis control application, one way to minimize sensory adaptation is to enable the biofeedback only when high accuracy control of the prosthetic leg is needed, such as facing terrain changes or experiencing scuffing. Robotic prosthetic legs have shown potential to detect such situations reliably [58].

Our biofeedback system also has potential to be used as a training tool to help amputees attain proficiency using the dEMG interface for prosthetic leg control. Previous upper limb studies show EMG feedback serves as an effective training tool. EMG feedback improves performance in a force matching task and this improvement is retained after the feedback is removed [59]. Further study should explore the potential of this biofeedback system for training.

The concept of our present EMG biofeedback system is closely related to that of the AMI enabled dEMG prosthesis control [14], except that we used sensory substitution to replace the missing sensation of muscle state with tactile stimulation. The benefit of our approach is that it is non-invasive and is potentially more accessible. However, the sensory substitution is less intuitive, requiring additional training for the users to re-establish the relationship between the perceived haptic sensation and muscle activity. In addition, AMI potentially enables the perception of the mechanical reciprocal relationship between agonist and antagonist muscles, while our approach allows the sensation of neuromuscular control of two muscles. Future studies should examine the trade-off between these two methods to enable effective translation of the technology to the lower limb amputee population.

One observation in this study was that EMG biofeedback had more impact on the TA to improve accuracy in matching DF positions than on the GAS for PF position matching. Such a muscle-specific effect was also observed in the previous studies in transtibial amputees [60], [61]. There are several potential explanations. First, evidence has shown that the TA has a stronger connection with the motor cortex compared to the GAS. The cortical control of TA enables accurate ankle motion adjustments during the swing phase for foot clearance [62]. The added feedback of TA activity may enable TB participants to voluntarily adjust TA control with more precision. Another explanation is pertinent to changes in muscle physiology that occur after a TB amputation. Recent studies found that for many TB individuals the motor unit properties of the TA is much more severely altered by amputation than those of the GAS [60]. For example, although the GAS of TB amputees generally follows the Henneman’s size principle [63], the TA has been observed to not consistently follow this same principle [60]. This change of muscle physiology indicates that amputees may need additional assistance to activate TA muscles appropriately. Finally, an additional possible explanation for why biofeedback was more helpful in DF than PF could be related to prosthetic socket design. Current socket design ensures a tight fit between the residual limb and socket. Since the GAS is much larger in volume than the TA, its contraction can lead to a greater change in volume of the residual limb which can alter the distribution of pressure within the socket [61]. In this case, pressure within the socket may serve as an additional source of biofeedback. The TA, being much smaller, would not have the same impact on pressure distribution, making EMG biofeedback for the TA much more necessary for improving the accuracy of control.

Between the two groups of participants recruited for this study, the ND group generally yielded more consistent and superior task performance than the TB group. This aligns with our expectations. In the ND group, the existence of intact muscle proprioception in the intact limbs provided additional sensory feedback to understand how to activate their muscles for ankle position control. In the other group, TB participants had impaired muscle proprioception in their residual limb due to limb amputation and muscle reorganization. As a result, TB participants showed large inter-person variation in task performance (Fig. 6). Both groups still saw significant improvements to overall accuracy in the BF condition, showing that our biofeedback system can be beneficial for improving the accuracy of dEMG control in both populations. In addition, our results suggest that amputees might benefit more from the biofeedback systems than ND.

One TB participant, TB06, was unable to accurately control the ankle position regardless of feedback condition. The large variation of TB amputees to control the residual ankle muscles have been also observed in other studies [16], [61], [64]. These differences may be caused by the variation in amputation surgeries, years of amputations, cause of amputations (e.g., diabetes versus traumas), post-amputation rehabilitation, etc. Perhaps in the future, researchers can build a predictive model based on these potential factors to predict a patient’s ability to coordinate the muscle activities for dEMG control of robotic prosthetic legs, if there are sufficient data collected from individuals with lower limb amputations.

Finally, we want to highlight one interesting observation of TB06. Even though TB06 performed poorly in the ankle position matching task both with and without EMG biofeedback, EMG biofeedback did improve TB06 muscle activity as shown in Fig. 7. Under the BF condition, there was a clear reduction in the co-activation between the GAS and TA muscles across the different targets. This shows that the EMG biofeedback was able to aid TB06 in differentiating between the two muscle groups. Although TB06 clearly still struggled to differentiate between individual targets, this reduction in co-activation further showcased the benefits of this EMG biofeedback strategy. It is possible that this participant needs a longer time to practice residual muscle activation and coordination with EMG biofeedback before being able to successfully control the robotic prosthetic ankle.

This study has limitations, which also lead to potential future work. Although our analysis leads to statistical significance with a small number of TB participants when we compare BF and NBF conditions, a larger number of individuals with limb amputation should be recruited and evaluated in the future to demonstrate the effects of our biofeedback system. In addition, the evaluation in this study was limited to the closed-loop operation of ankle prosthesis via EMG biofeedback and dEMG control to match the desired joint position. Like we mentioned previously, we should extend the design and evaluation to closed-loop operation to other prosthetic joint mechanics, such as torque or impedance, and extend the evaluation task to dynamic tasks, such as walking. These will be our future work.

## V. Conclusion

In this study we investigated the application of EMG-based biofeedback to restore closed-loop neural control of a powered prosthetic ankle. We developed a biofeedback system, composed of a HD haptic vest and an encoder that mapped EMG magnitude of residual ankle muscles to tactile vibration patterns. The effectiveness of this biofeedback system was demonstrated in a position matching task performed by both ND participants and TB participants. Our results found that after a brief acclimation procedure, in which participants were familiarized with the feedback and control strategy, both groups were able to perform the position matching task more accurately when biofeedback was provided. For TB participants, the feedback led to more separatable EMG control signals and a clear improvement in performance when they tried to match dorsiflexion targets. At the same time, the variation among amputees was high. The results presented in this study show that EMG biofeedback has the potential to be a powerful tool to help amputees attain proficiency using the dEMG control interface without an invasive surgical procedure. In future work, we will evaluate the impact of this biofeedback platform during locomotive tasks when user control goals are beyond position matching.

## Supplementary Material

supp1-3691653

## Figures and Tables

**TABLE I:** TB Participant Demographics

ID	Sex	Age	Weight (kg)	Height (m)	Time Since Amputation (years)	Cause of Amputation	K-Level
TB01	M	55	99.8	1.83	10	Trauma	4
TB02	F	67	117.0	1.67	2	Diabetes	3
TB03	M	54	104.2	1.68	24	Trauma	3
TB04	M	33	117.9	1.73	21	Trauma	4
TB05	M	46	88.5	1.85	21	Trauma	3
TB06	M	58	106.6	1.78	8	Trauma	3
