# Supplementary material for: Using EMG Biofeedback to Restore Closed-Loop Neural Control on a Powered Prosthetic Ankle
Source: IEEE Trans Neural Syst Rehabil Eng. Author manuscript; Available in PMC 2026 Jun 28. (PMC13310372; doi:10.1109/TNSRE.2026.3691653)
Supplement: supp1-3691653 [file NIHMS2179447-supplement-supp1-3691653.pdf]

In this paper, silhouette scores were calculated to quantify TB participant's ability to generate distinct EMG signals in order to reach different postures. Silhouette scores act as a measurement of how similar an observed data point is to its own cluster versus other clusters, and are calculated using the following equation:

$$s(i) = \frac{b(i) - a(i)}{\max(a(i), b(i))}$$

Where i is the observed data point, $a(i)$ is the mean distance between i and each other observation in the same cluster, and $b(i)$ is the mean distance between i and each observation in the neighboring cluster. Here, the "neighboring cluster" is defined as the cluster, of which i is not a member, with the minimum mean distance between each of its observations and the observation i.

An illustrated example is presented below in Fig. 1.

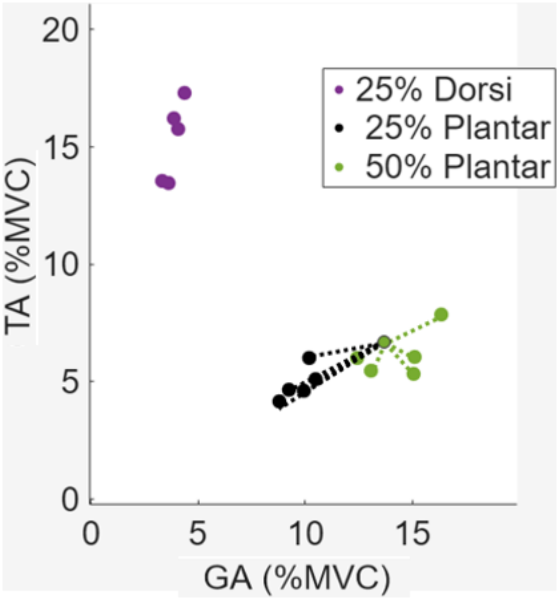

Fig. 1. Example of silhouette score calculation for an observation from when a participant was prompted to move to "50% Plantarflexion"

In this example $a(i)$ is defined as the mean distance between the observation i and each other observation in the 50% plantarflexion cluster. Then, $b(i)$ is defined as the mean distance between the observation i and each observation in the neighboring cluster, in this case 25% dorsiflexion. Since $b(i)$ appears to be larger than $a(i)$ the silhouette score will be positive and close to 1. This shows that i was properly clustered, and that the participant was able to generate an EMG signal that was both similar to their other attempts at reaching for 50% plantarflexion and distinct from their attempts to reach for other targets.

If the silhouette score was close to -1, i would be closer to the 25% plantarflexion cluster than the 50% plantarflexion cluster.
